# Supplementary material for: Structure of a membrane-bound menaquinol:organohalide oxidoreductase
Source: Nat Commun. 2023 Nov 3;14:7038. doi: 10.1038/s41467-023-42927-7 (PMC10624902; doi:10.1038/s41467-023-42927-7)
Supplement: Supplementary file 1 — Supplementary Information [file 41467_2023_42927_MOESM1_ESM.pdf]

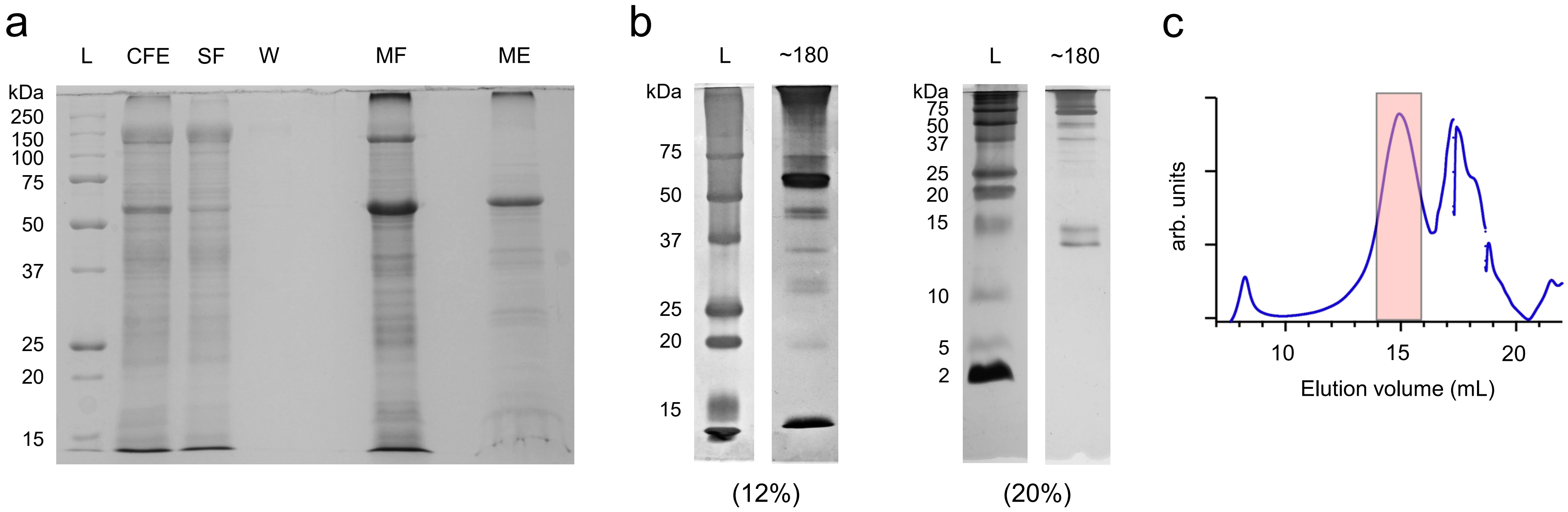

Supplementary Figure 1 – Extraction of the RDase complex from *D. hafniense* strain TCE1. SDS-PAGE of **a**, Representative protein samples obtained during the extraction the complex (out of 4 experiments with similar results), and of **b**, 2D-electrophoresis of the ~180 kDa band in 12% and 20% acrylamide gels. **c**, Elution profile from the size-exclusion chromatographic analysis of the membrane extract (the red box indicates the fractions collected for cryo-EM analysis). Legend: L: protein ladder; CFE: cell-free extract; SF: soluble fraction; W: wash sample; MF: membrane fraction; ME: membrane extract. Source data are provided as a Source Data file.

a

	Total spectrum count (% coverage)
PceA	2935 (84)
PceB	22 (41)

b

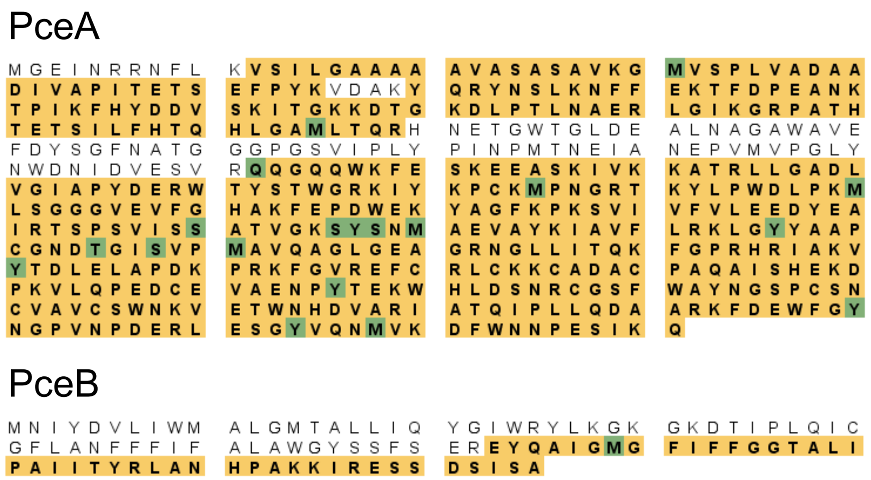

Supplementary Figure 2 - LC-MS/MS analysis of the ~180 kDa CN-PAGE band. **a**, Total spectrum count of PceA and PceB peptides. **b**, Representative coverage of PceA and PceB proteins highlighted in colour. Green residues indicate amino acids that were found post-translationally modified for a certain number of the detected peptides (oxidation for Met; phosphorylation for Ser, Thr and Tyr).

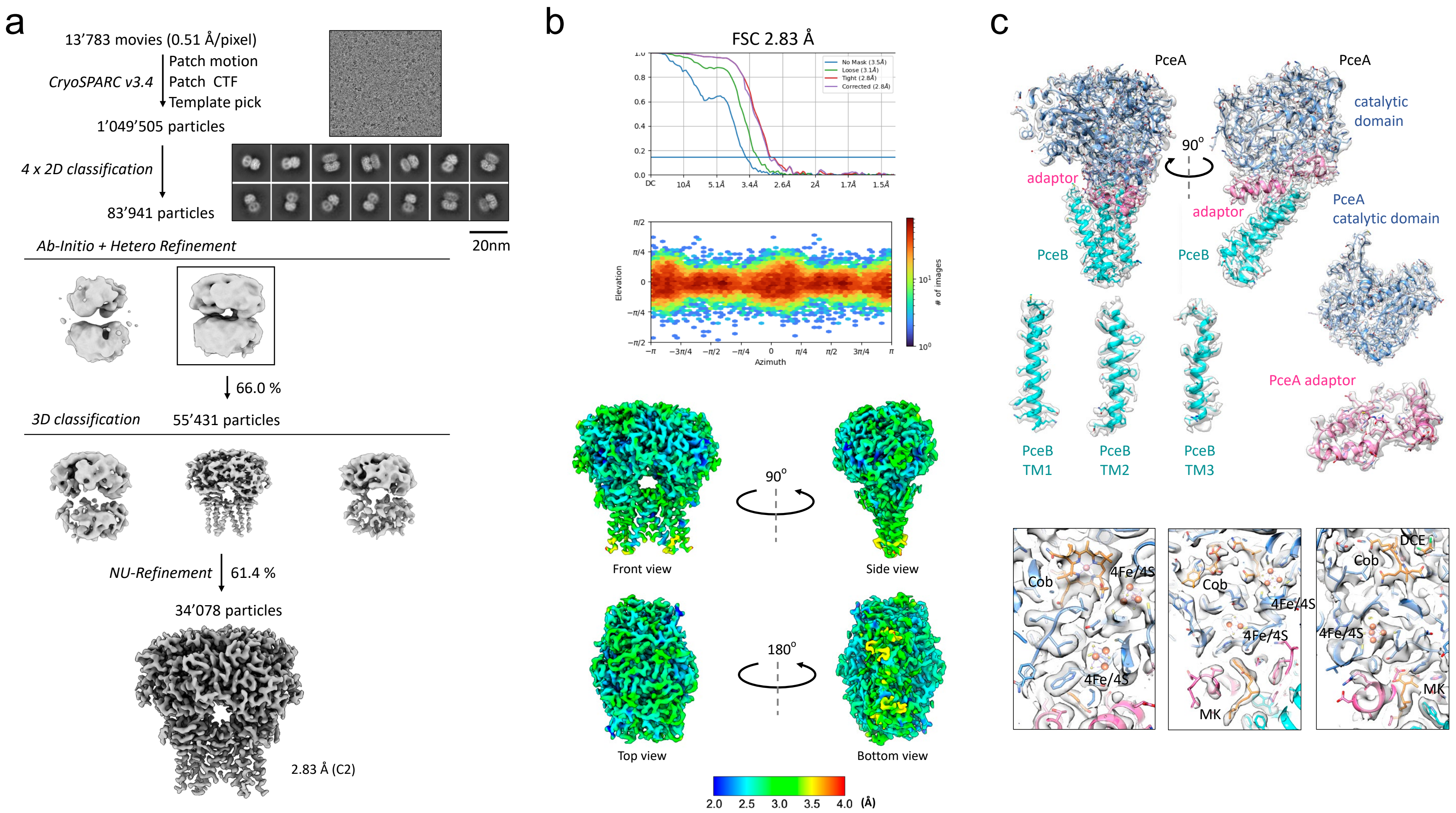

Supplementary Figure 3 - Cryo-EM analysis of the $PceA_2B_2$ complex. **a**, Flow chart of the cryo-EM data analysis. **b**, Fourier-Shell Correlation (FSC) curve indicating an overall resolution of 2.83 Å (FSC 0.143), direction distribution plot and global refined map coloured by local resolution are given in different views. **c**, Density maps and structure interpretation of PceA and PceB proteins, and of the cofactors.

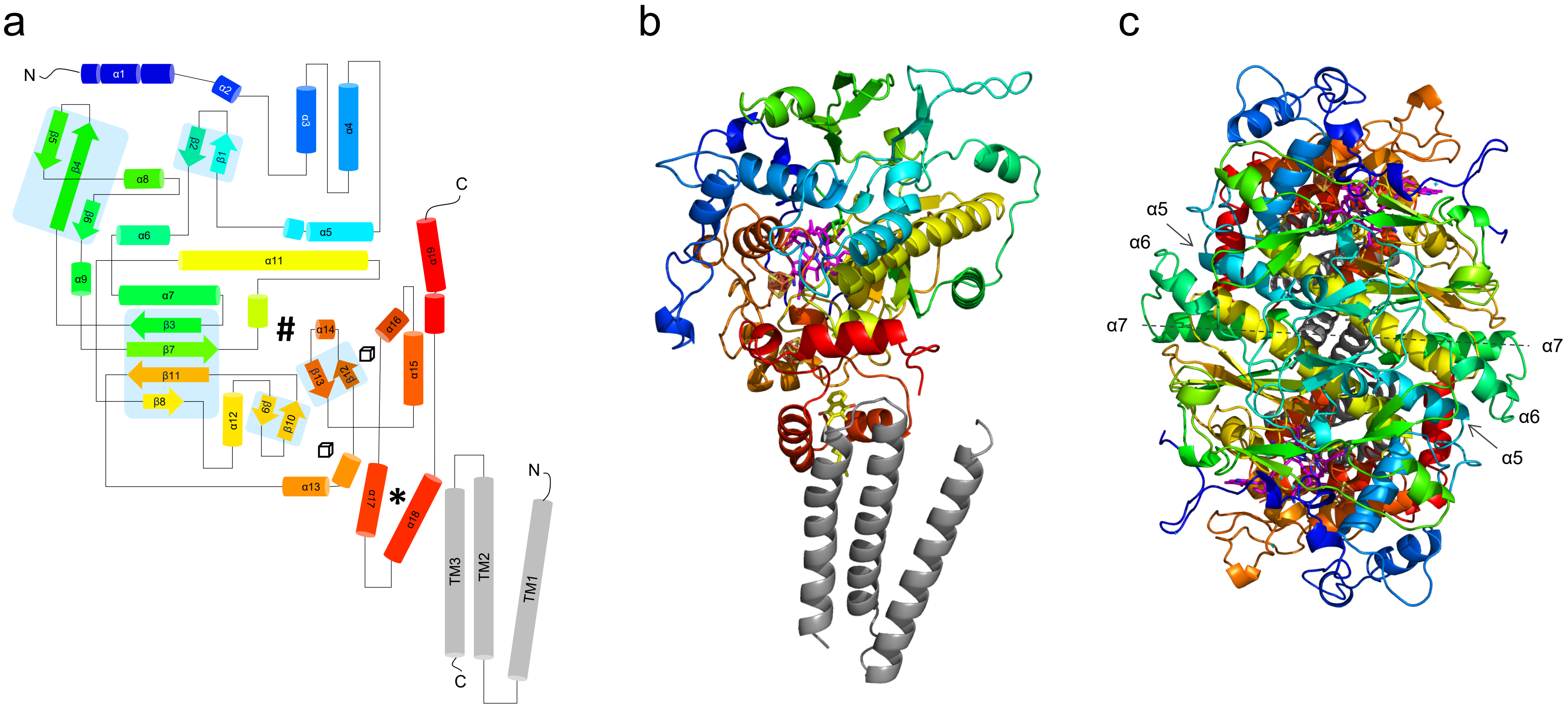

Supplementary Figure 4 - Topology analysis of DhPceA$_2$B$_2$ secondary structures. **a**, Topology diagram of the DhPceAB heterodimer depicted in rainbow colour mode. The hash sign, the cubes and the asterisk indicate the position of the cofactors in the PceAB dimer: the cobalamin, both [4Fe-4S] clusters and the menaquinone, respectively. **b**, Front view of the corresponding cartoon structure. **c**, Top view of the DhPceA$_2$ cartoon structure.

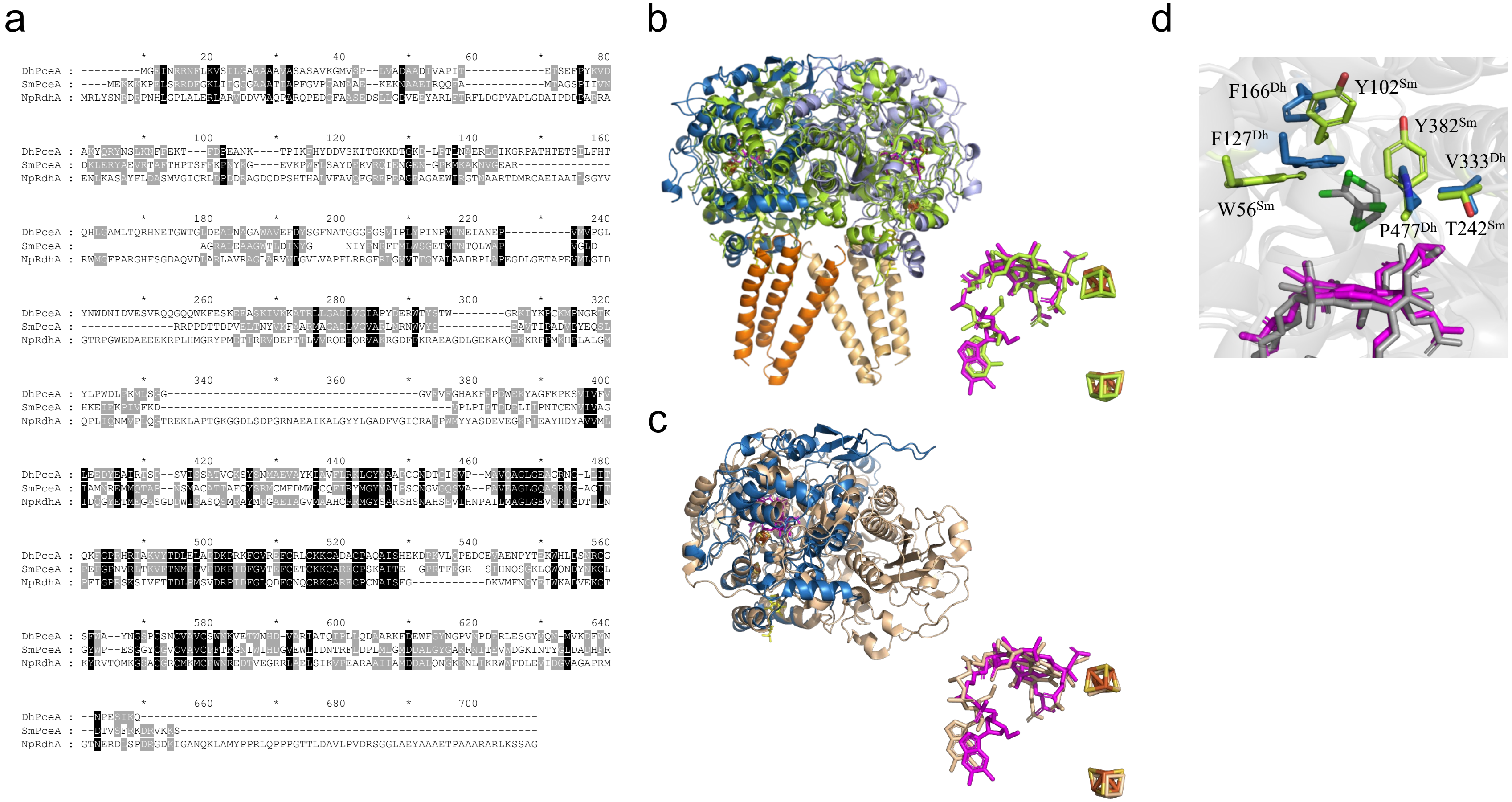

Supplementary Figure 5 - Sequence and structure alignments of reductive dehalogenases. **a**, Primary sequence alignment of DhPceA, SmPceA and NpRdhA. Conserved amino acids are shaded in black. **b**, Structure alignment of DhPceA$_2$B$_2$ and SmPceA$_2$ depicted in front view. The superimposition of cofactors is also shown. **c**, Structure alignment of DhPceA and NpRdhA. The cofactors are also shown in a separate panel. **d**, Active site residue differences between DhPceA and SmPceA. <u>Colour code</u>: DhPceA$_2$B$_2$ is displayed with the same colours as before, SmPceA$_2$ and its cofactors are in green, while NpRdhA and cofactors in cream. <u>Legend</u>: Dh, *Desulfitobacterium hafniense*; Sm, *Sulfurospirillum multivorans*; Np, *Nitratireductor pacificus*.

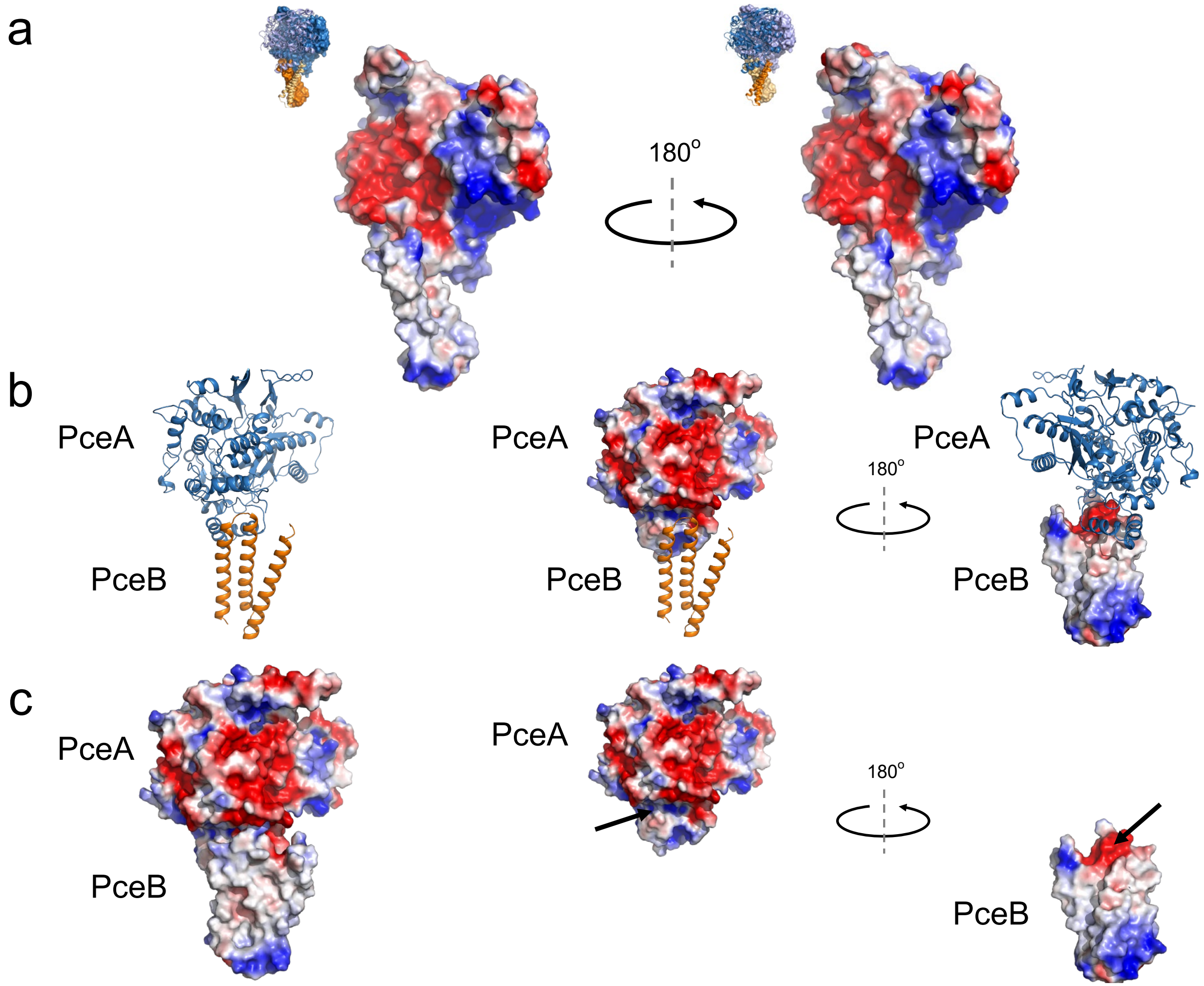

Supplementary Figure 6 - Surface interactions within the $PceA_2B_2$ complex. **a**, Electrostatic interactions between both PceA monomers highlighting the opposite overall charges in the contact area, and hydrophobic interactions between both PceB monomers.
b and **c**, Electrostatic interactions between PceA and PceB subunits in each PceAB heterodimer.

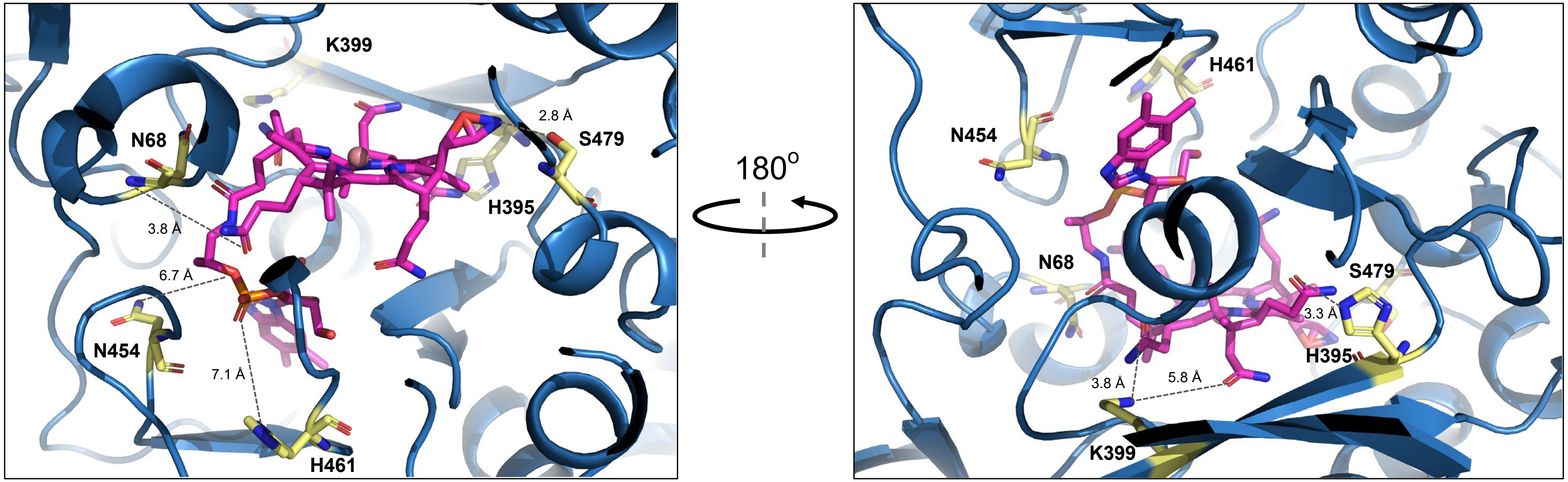

Supplementary Figure 7 - Structural insights in the cobalamin binding site of DhPceA showing amino acids at H-bond distance to the cobalamin cofactor.

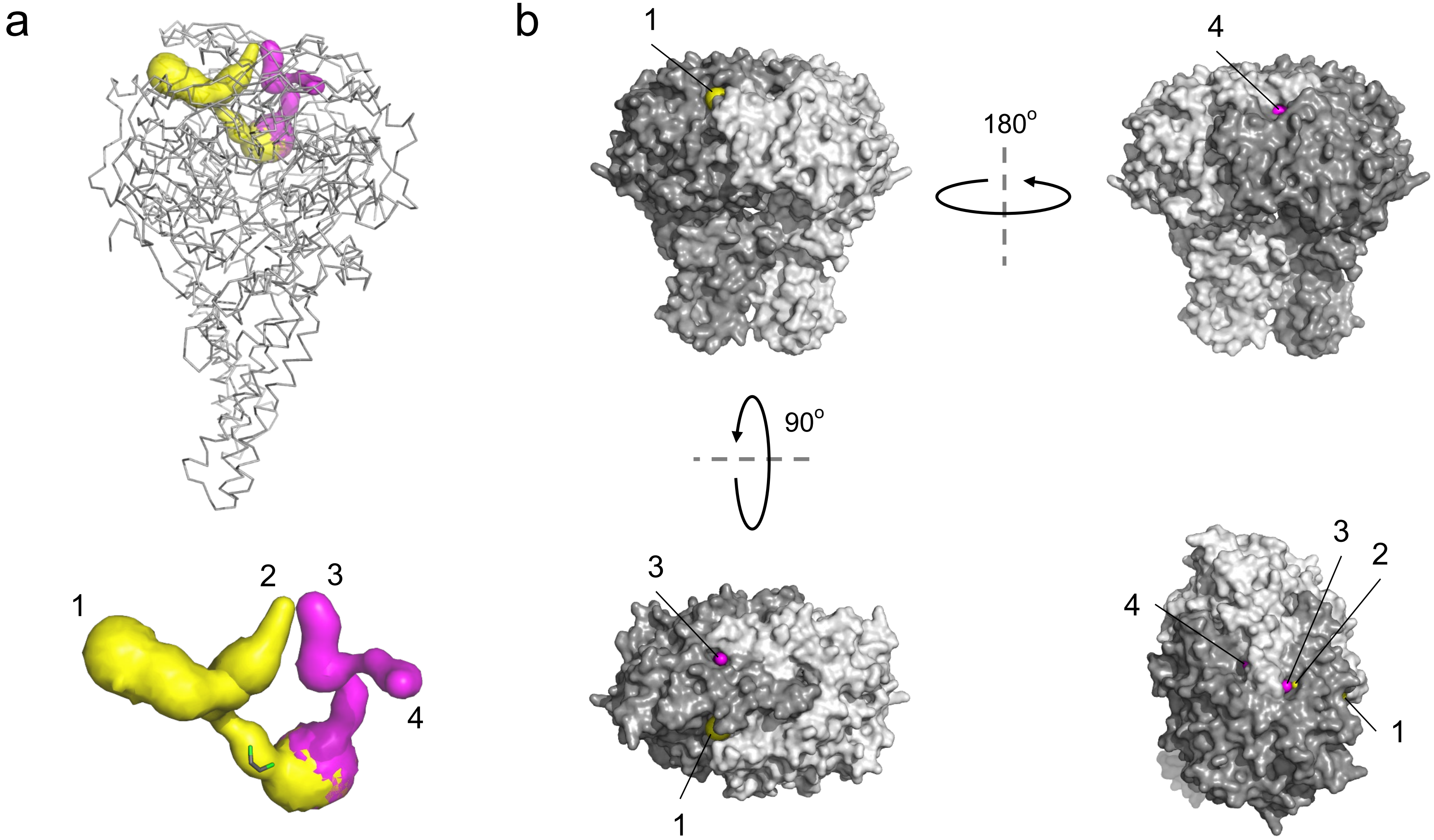

Supplementary Figure 8 - Channel prediction in DhPceA. **a**, Overview of both channels connecting the active site to the outside solution (see also Figure 4 in the article). **b**, Position of the channel exits at the surface of the DhPceA$_2$B$_2$ complex. For clarity, the channel exits are only indicated for one PceA subunit (depicted in dark grey).

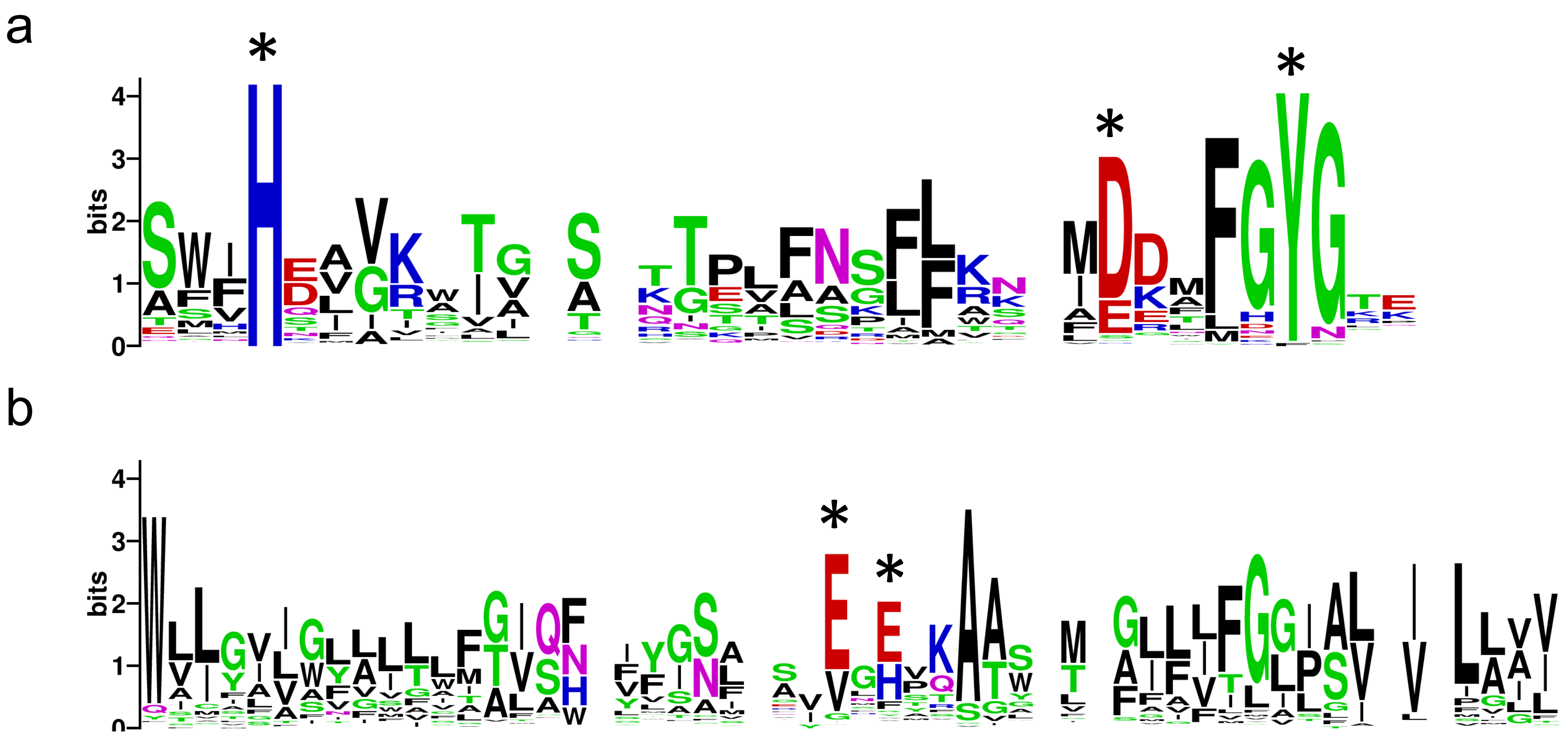

Supplementary Figure 9 - Key conserved residues in the menaquinone-binding pocket of RdhAB heterodimers. Weblogo representation of selected regions of **a**, RdhA and **b**, RdhB sequence alignments. Stars indicate in the C-terminal region of RdhA sequences a fully conserved histidine residue (H495 in DhPceA) and an almost fully conserved tyrosine (Y520), as well as a highly conserved negatively charged residue (D515). The loop connecting transmembrane helices 2 and 3 in RdhB sequences harbours highly conserved glutamate residues (E61 and E63 in DhPceB). Legend: Dh, *Desulfitobacterium hafniense*.

Supplementary Table 1 - Cryo-EM data collection, refinement and validation statistics.

	MOOR complex ($PceA_2B_2$) (EMDB-18148) (PDB 8Q4H) (EMPIAR-11719)
**Data collection and processing**	
Magnification	155kx (normal)
Voltage (kV)	300
Electron exposure (e–/Å^2)	60
Defocus range (-µm)	1.0 -2.2
Pixel size (Å)	0.51
Symmetry imposed	C2
Initial particle images (no.)	1 049 505
Final particle images (no.)	34 078
Map resolution (Å)	2.83
FSC threshold	0.143
Map resolution range (Å)	30-2.0
**Refinement**	
Initial model used (PDB code)	n/a
Model resolution (Å)	3.0
FSC threshold	0.143
Model resolution range (Å)	30-3.0
Map sharpening *B* factor (Å^2)	-63
Model composition	
Non-hydrogen atoms	9510
Protein residues	1184
Ligands	DCE: 2
	SF4: 4
	MQ7: 2
	COB: 2
B factors (Å^2)	
Protein	14.07/88.61/35.26
Ligand	22.0/30.78/27.39
R.m.s. deviations	
Bond lengths (Å)	0.004 (4)
Bond angles (°)	0.574 (6)
Validation	
MolProbity score	1.32
Clashscore	5.82
Poor rotamers (%)	0.00
Ramachandran plot	
Favored (%)	98.38
Allowed (%)	1.45
Disallowed (%)	0.17
